# Supplementary material for: A selective ER‐phagy exerts procollagen quality control via a Calnexin‐FAM134B complex
Source: EMBO J. 2018 Dec 17;38(2):e99847. doi: 10.15252/embj.201899847 (PMC6331724; doi:10.15252/embj.201899847)
Supplement: Supplementary file 1 — Expanded View Figures PDF [file EMBJ-38-e99847-s001.pdf]

Expanded View Figures

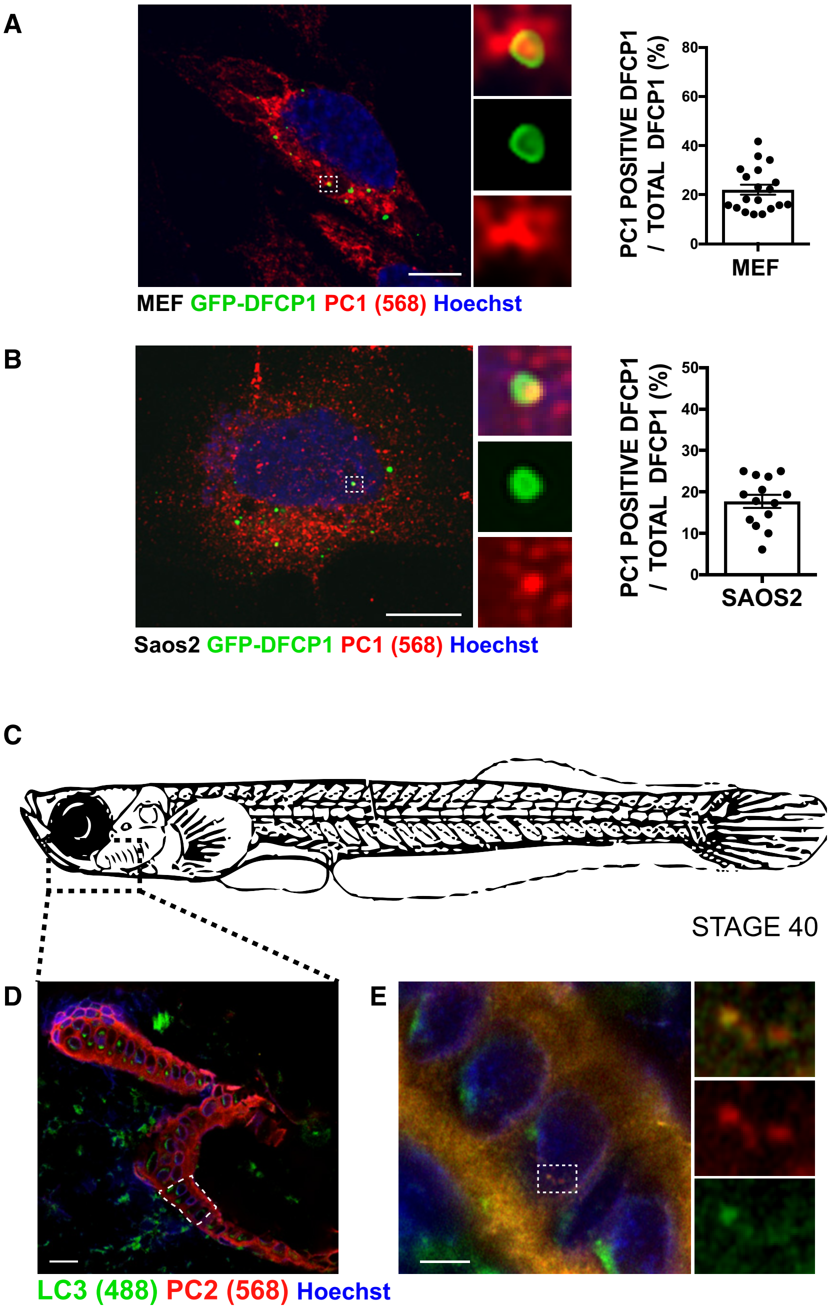

Figure EV1. PC is an autophagy substrate.

A, B Scanning confocal analysis of immunofluorescence for PC1 (568, red) and GFP-DFCP1 (green) in (A) MEF and (B) Saos2 and quantification of AVs positive for GFP-DFCP1 containing PC1 expressed as % of total DFCP1 per cell (mean ± SEM). The insets show higher magnification (A = x5.9; B = x6.04) and single colour channels of the boxed area. Scale bar = 10 µm. (A) n = 19 and (B) n = 14 cells counted per condition; three independent experiments.

C Schematic representation of a stage 40 medaka fish. Dotted box represents area of mandible analysed in (D and E).

D Scanning confocal image of mandible from stage 40 medaka, immunostained with LC3 (488, green) PC2 (568, red) and nuclei stained with Hoechst (blue). Dotted box represents area of mandible containing osteoblasts that was further analysed in (E). Scale bar = 20 µm.

E Airyscan confocal image of mandible at higher magnification, scale bar = 3 µm. Boxes on the right show magnification (x4.02) of boxed area.

© 2018 The Authors

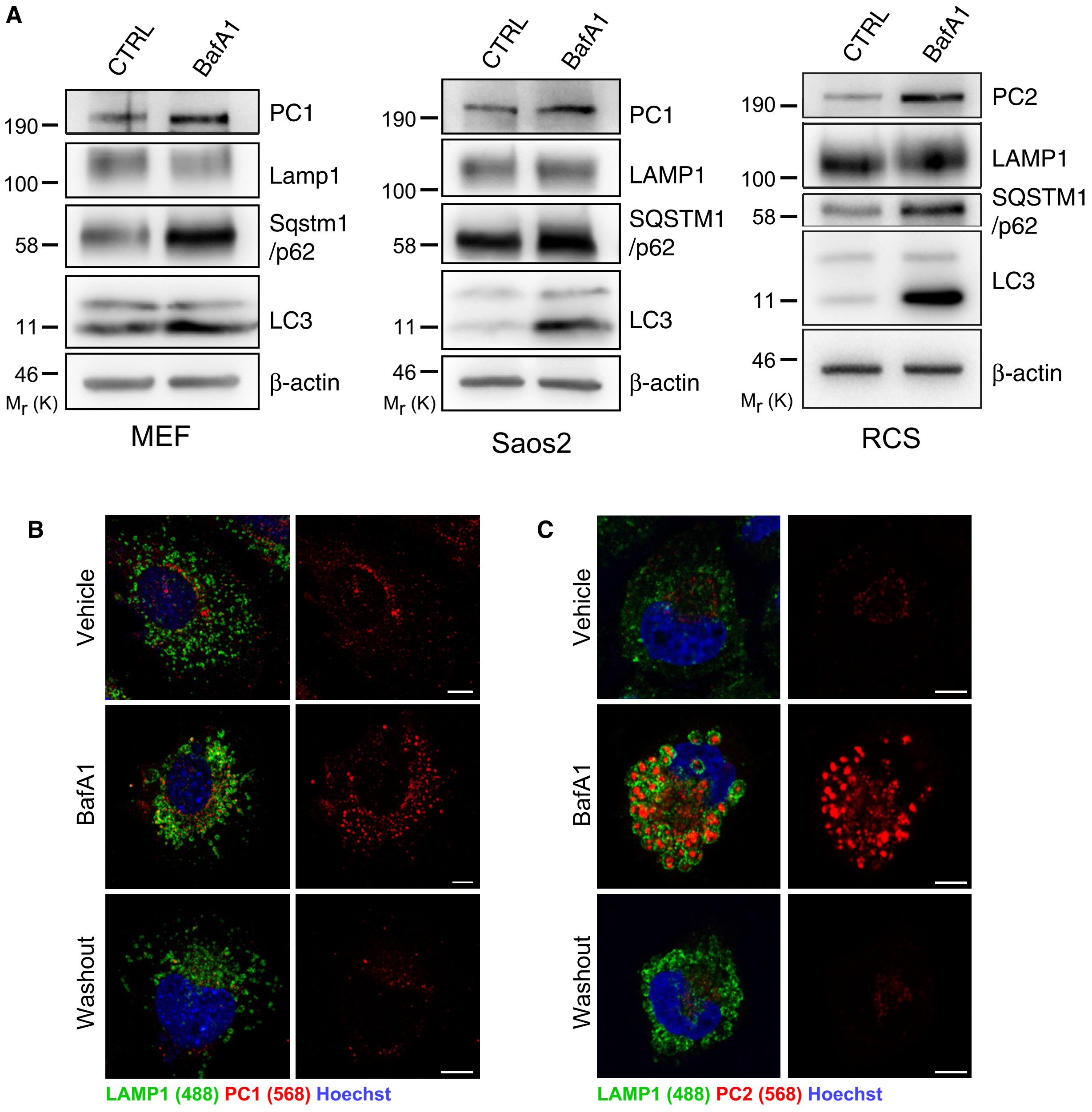

Figure EV2. PC1 and PC2 are autophagy substrates that are degraded in the lysosome.

A MEFs, Saos2 and RCS were untreated or treated with 100 nM BafA1 for 6 h in MEFs, 100 nM BafA1 for 9 h in Saos2, 200 nM BafA1 for 6 h in RCS, then lysed and analysed by Western blot. Bands were visualized with antibodies against PC1, PC2, LAMP1, SQSTM1/p62, LC3 and β-actin. Western blots are representative of three independent experiments.

B, C (B) MEFs or (C) RCS treated with vehicle, 100 nM BafA1 for 4 h, followed by 4-h washout. Cells immunolabelled with LAMP1 (488, green) and PC1 (568, red). Nuclei were stained with Hoechst (blue). Scale bars = 10 μm.

Source data are available online for this figure.

© 2018 The Authors

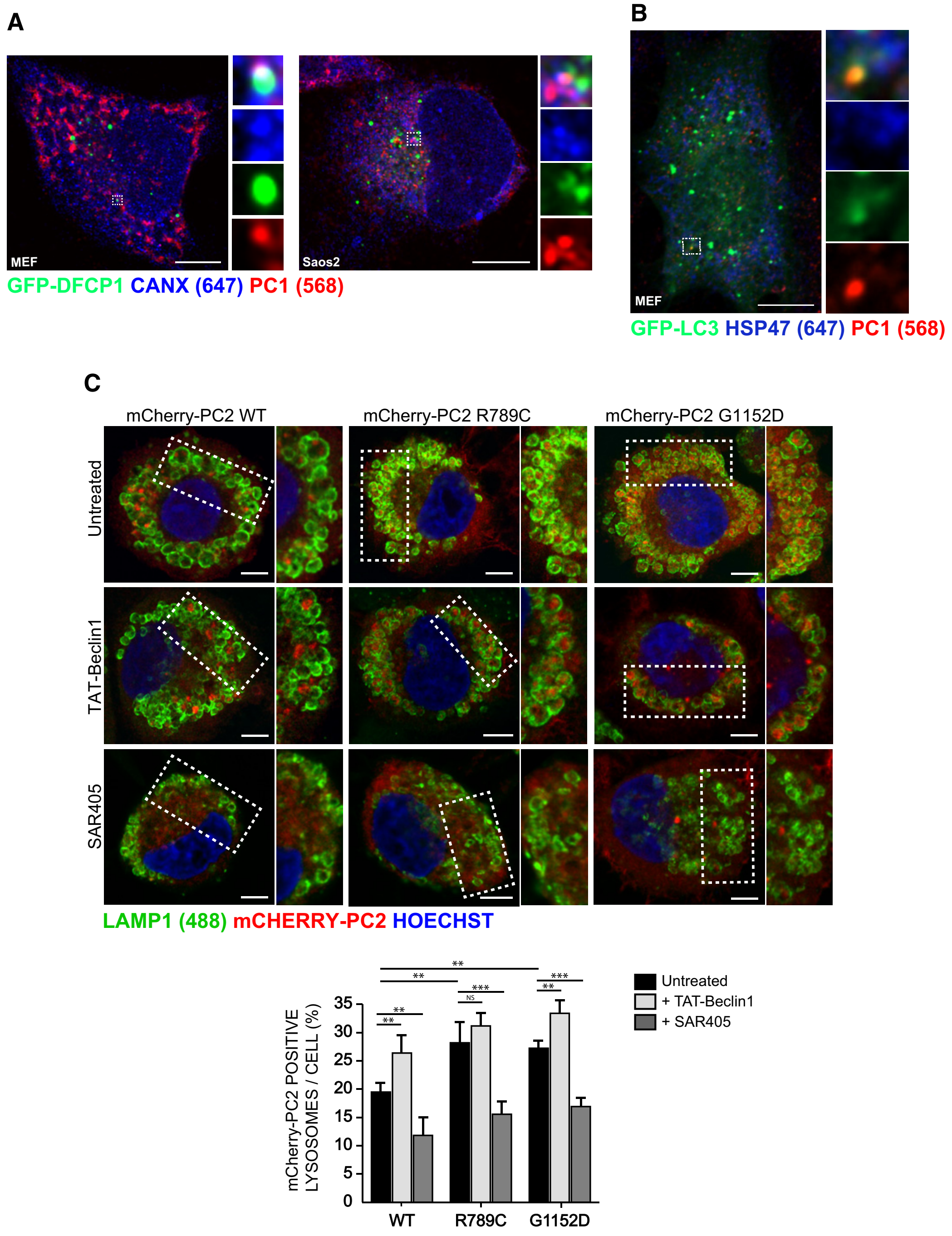

Figure EV3. Misfolded PC in the ER is targeted to lysosomes via autophagy.

A DFCP1, a marker of autophagosome biogenesis co-localizes with PC1 and CANX. GFP-DFCP1 (green) expressing MEF and Saos2 immunolabelled for PC1 (568, red) and CANX (647, blue). Cells were imaged with scanning confocal microscopy. The insets show higher magnification (left = x5.57; right = x4.25) and single colour channels of the boxed area. Scale bars = 10 μm.

B Hsp47 is excluded from PC1 containing autophagosomes. GFP-LC3 (green) expressing MEFs immunolabelled for PC1 (568, red) and Hsp47 (647, blue). Cells were imaged by scanning confocal microscopy. The insets show higher magnification (x4.34) and single colour channels of the boxed area. Scale bars = 10 μm.

C Mutant PC2 is targeted to lysosomes at a higher rate than WT, and modulated via autophagy. RCS cells were transiently transfected with mCherry-PC2 WT, R789C or G1152D mutants (568, red) and treated for 6 h with 100 nM BafA1, and as indicated with SAR405 or Tat-BECLIN-1. Cells were fixed and immunolabelled for LAMP1 (488, green). Nuclei were stained with Hoechst (blue). The insets show higher magnification (top left to bottom right: x1.37, x1.2, x1.39, x1.34, x1.44, x1.07, x1.61, x1.7, x1.56). Scale bars = 10 μm. Bar graph shows quantification of LAMP1 vesicles positive for mCherry-PC2, expressed as % of total lysosomes per cell (mean ± SEM), minimum of n = 11 cells per genotype. Two-way ANOVA with Tukey's *post hoc* test performed and *P*-value adjusted for multiple comparisons. ns ≥ 0.05, $^{**}P < 0.005$; $^{***}P < 0.0001$.

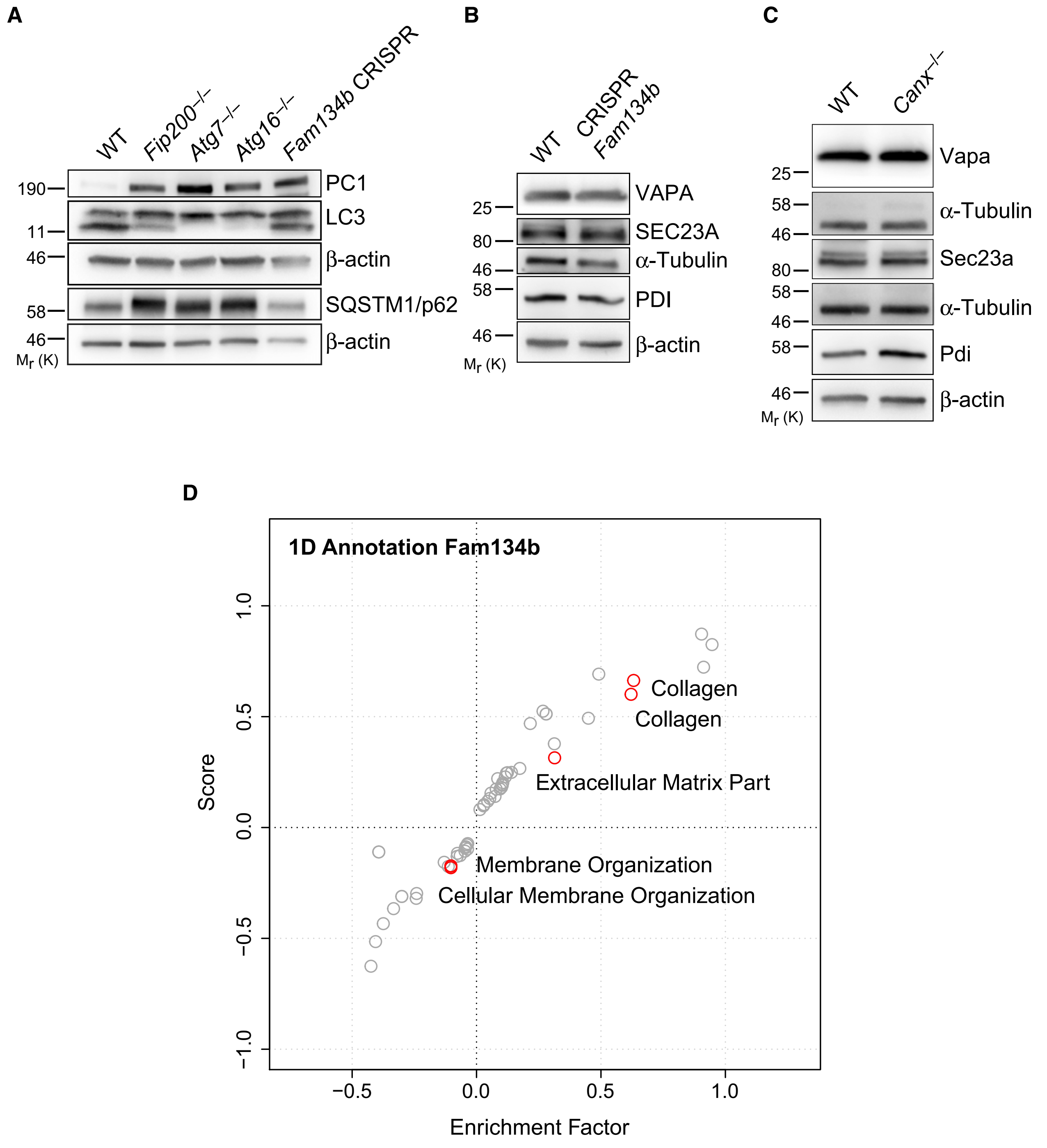

Figure EV4. FAM134B and CANX deficiency fails to increase accumulation of other ER-resident proteins.

A–C WT and KO MEFs for indicated genes were lysed at steady state and analysed by Western blotting. Bands were visualized with antibodies against PC1, SQSTM1/p62, LC3, Sec23a, VAPA, PDI and α-tubulin or β-actin as controls. Western blots are representative of three independent experiments.

D Collagens are highly enriched in *Fam134b*$^{-/-}$. Scatter plot for 1D annotation enrichment analysis of significantly upregulated peptides in the *Fam134b*$^{-/-}$ MEFs proteome.

Source data are available online for this figure.

 © 2018 The Authors

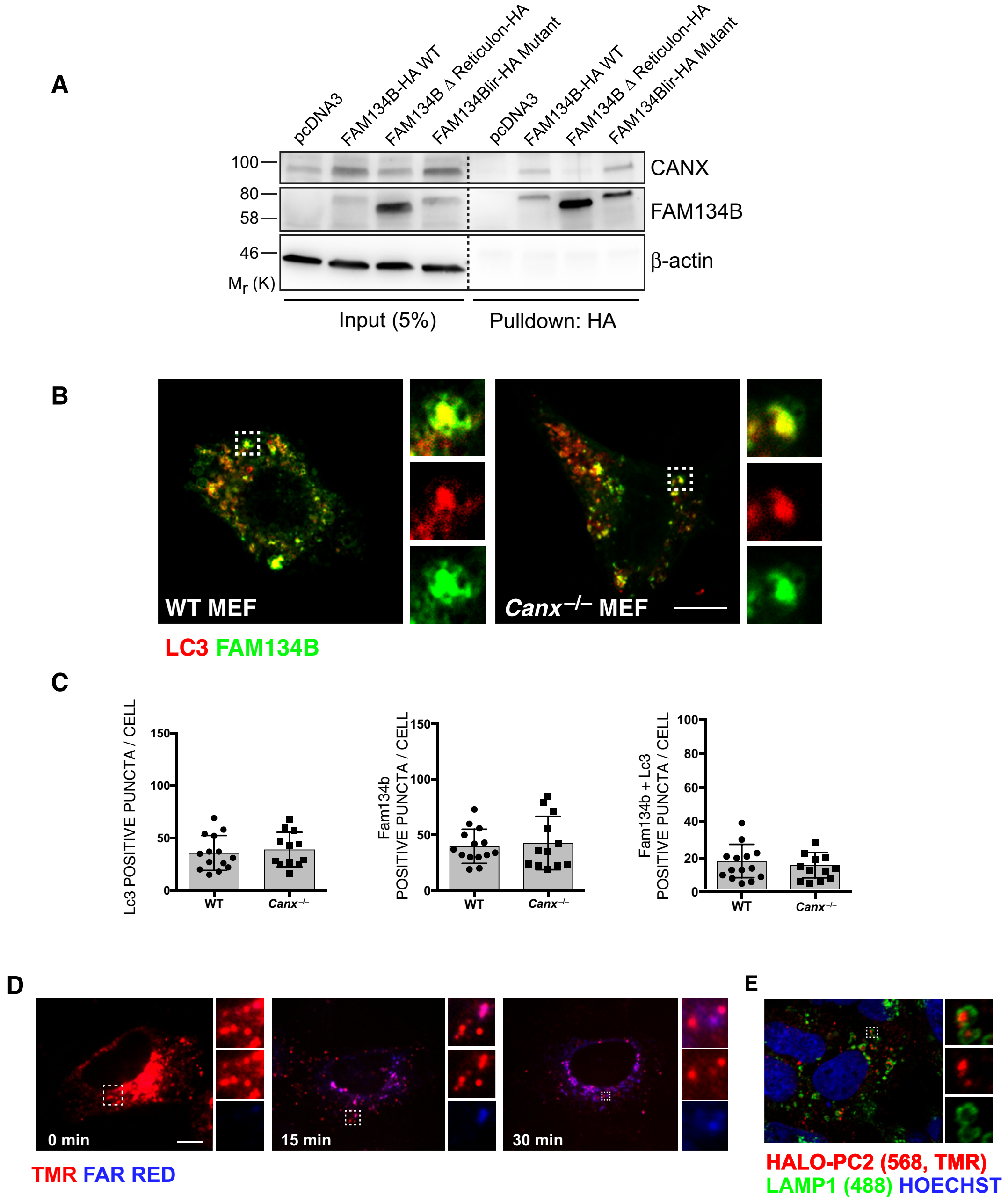

Figure EV5. FAM134B-CANX interaction is not modulated by PC.

A HeLa (Kyoto) cells were transfected with empty vector control, FAM134B-HA WT or mutant constructs as indicated. Complexes were immunoisolated with HA-magnetic beads, separated by Western blot and visualized with antibodies against CANX, FAM134B and β-actin as control. 5% of the input is shown. Western blots are representative of three independent experiments.

B, C WT and *Canx*$^{-/-}$ MEFs were transiently transfected with RFP-LC3 and WT GFP-FAM134B. Representative immunofluorescence, the insets show higher magnification (x3.62) and single colour channels of the boxed area. Scale bar = 10 μm. Quantification of AVs positive for RFP-LC3 (red) containing GFP-FAM134B (green) expressed as % of total LC3, FAM134B and FAM134B+LC3 per cells (mean ± SEM). n = 12 cells counted per condition; three independent experiments.

D, E Addition of HaloTag does not perturb trafficking of PC2 molecules. (D) Pulse chase of U2OS cells transfected with Mifepristone inducible HALO-PC2. Cells were pulsed for 20 min with HALO ligand (568, TMR, red) then chased in medium containing HALO ligand (Far red, blue) for 0, 15, 30 min. Images show that after 30 min, the majority of TMR-bound HALO was secreted. The insets show higher magnification (left = x2.56, middle = x3.31, right = x6.79) and single colour channels of the boxed area. Scale bar = 10 μm. (E) Scanning confocal analysis of U2OS transfected with HALO-PC2, treated for 9 h with 100 nM BafA1 in the presence of TMR (red) and immunolabelled for LAMP1 (488, green). Nuclei were stained with Hoechst (blue). The insets show higher magnification (x4.52) and single colour channels of the boxed area. Scale bar = 10 μm.

Source data are available online for this figure.

© 2018 The Authors
