# Supplementary material for: A selective ER‐phagy exerts procollagen quality control via a Calnexin‐FAM134B complex
Source: EMBO J. 2018 Dec 17;38(2):e99847. doi: 10.15252/embj.201899847 (PMC6331724; doi:10.15252/embj.201899847)
Supplement: Supplementary file 3 — Movie EV1 [file EMBJ-38-e99847-s003.zip › MOVIE_EV1/Expanded_View_Movie_1.docx]

**Expanded View Movie 1.** U2OS cells transiently expressing GFP-LC3, mCHERRY-PC2 and RDEL-HALO (Far Red ligand, blue), after incubation at 40 °C for 3h and release to 32 °C. Movie starts when temperature arrives at 32 °C. Acquisition at 1 frame per second.
