# Supplementary material for: A selective ER‐phagy exerts procollagen quality control via a Calnexin‐FAM134B complex
Source: EMBO J. 2018 Dec 17;38(2):e99847. doi: 10.15252/embj.201899847 (PMC6331724; doi:10.15252/embj.201899847)
Supplement: Supplementary file 4 — Movie EV2 [file EMBJ-38-e99847-s004.zip › MOVIE_EV2/Expanded_View_Movie_2.docx]

**Expanded View Movie 2.** U2OS cells transiently expressing GFP-2-FYVE, mCHERRY-PC2 and RDEL-HALO (Far Red ligand, blue), after incubation at 40 °C for 3h and release to 32 °C. Movie starts 8 min after temperature arrival at 32 °C. Acquisition at 1 frame per second.
