# Supplementary figures and images for: A selective ER‐phagy exerts procollagen quality control via a Calnexin‐FAM134B complex
Source: EMBO J. 2018 Dec 17;38(2):e99847. doi: 10.15252/embj.201899847 (PMC6331724; doi:10.15252/embj.201899847)

Uncropped gels with size marker indications

EV FIGURE 2A

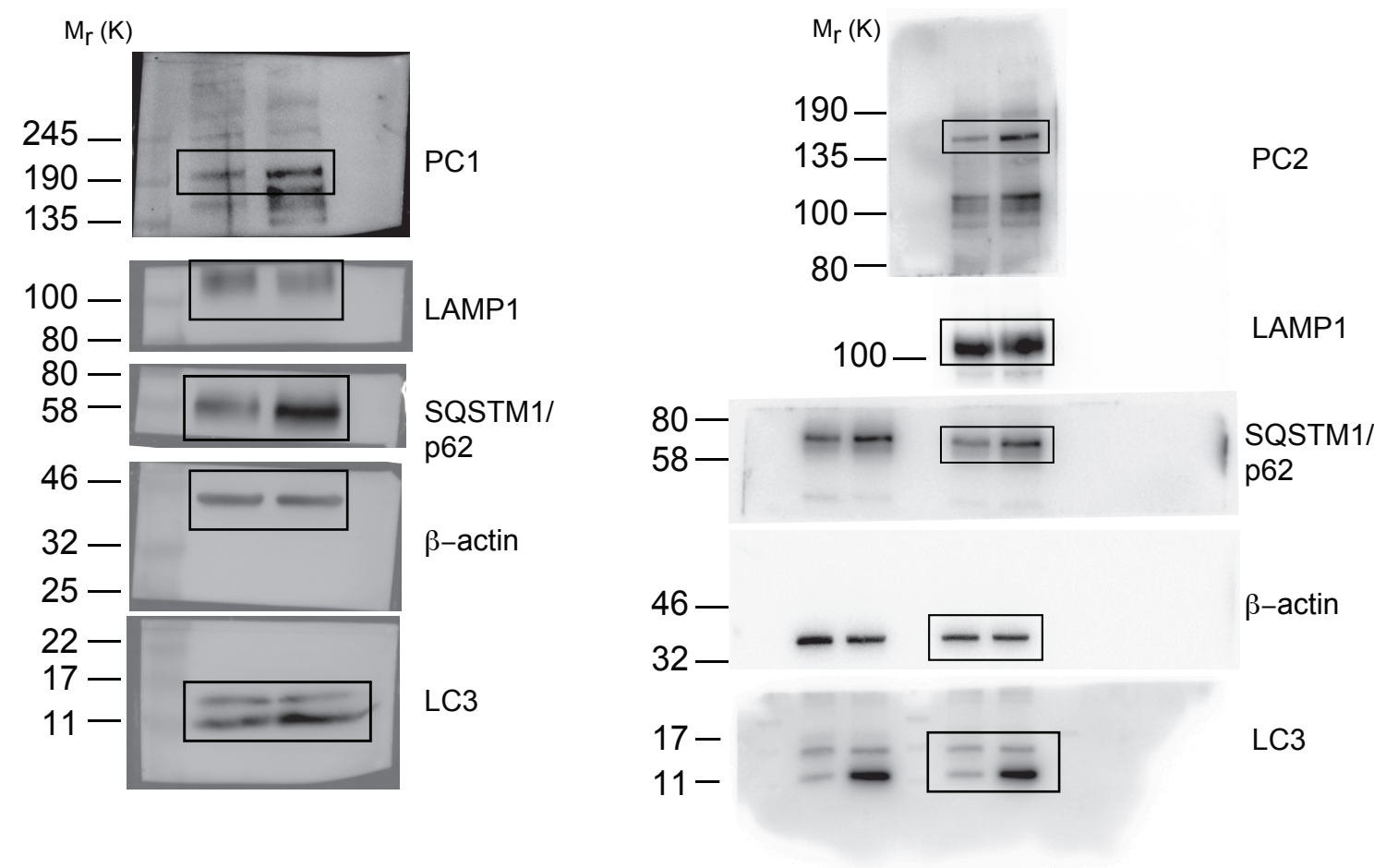

MEF

RCS

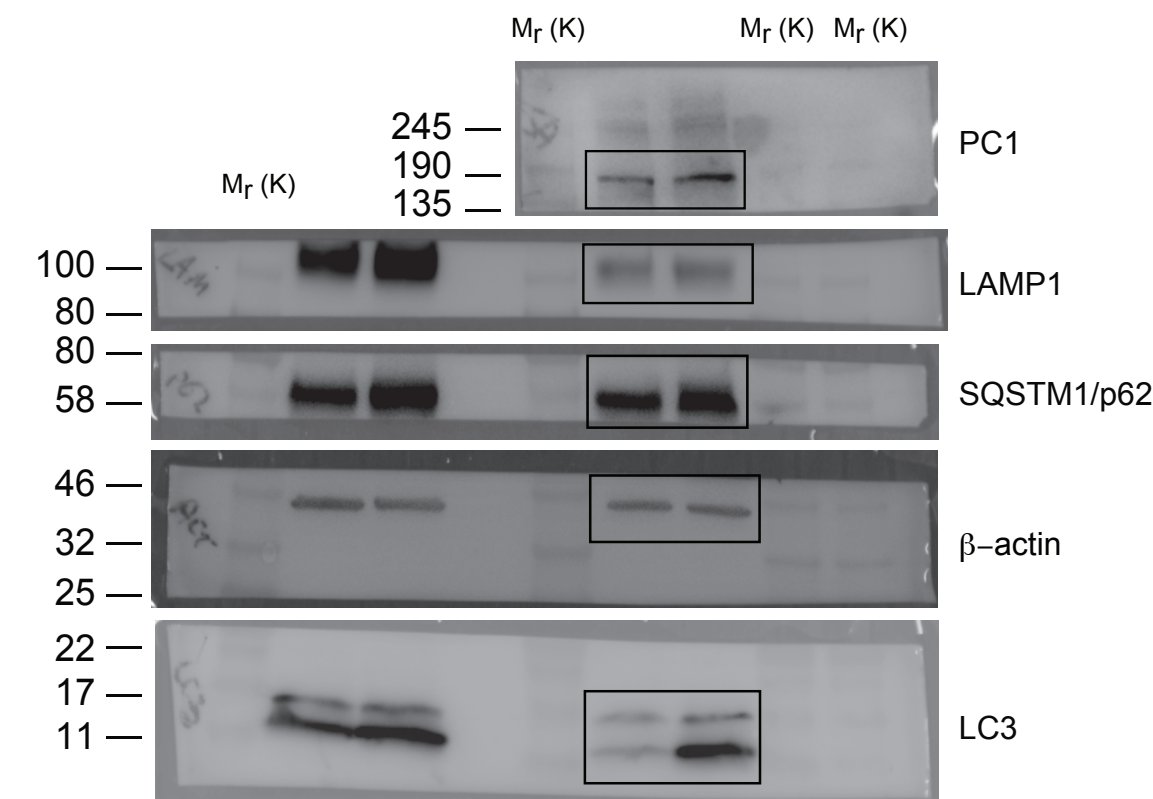

Saos2

Supplement: Supplementary file 5 — Source Data for Expanded View [file EMBJ-38-e99847-s005.zip › Figure_Source_Data_Fig_EV2.pdf]

Uncropped gels with size marker indications

EV FIGURE 4A

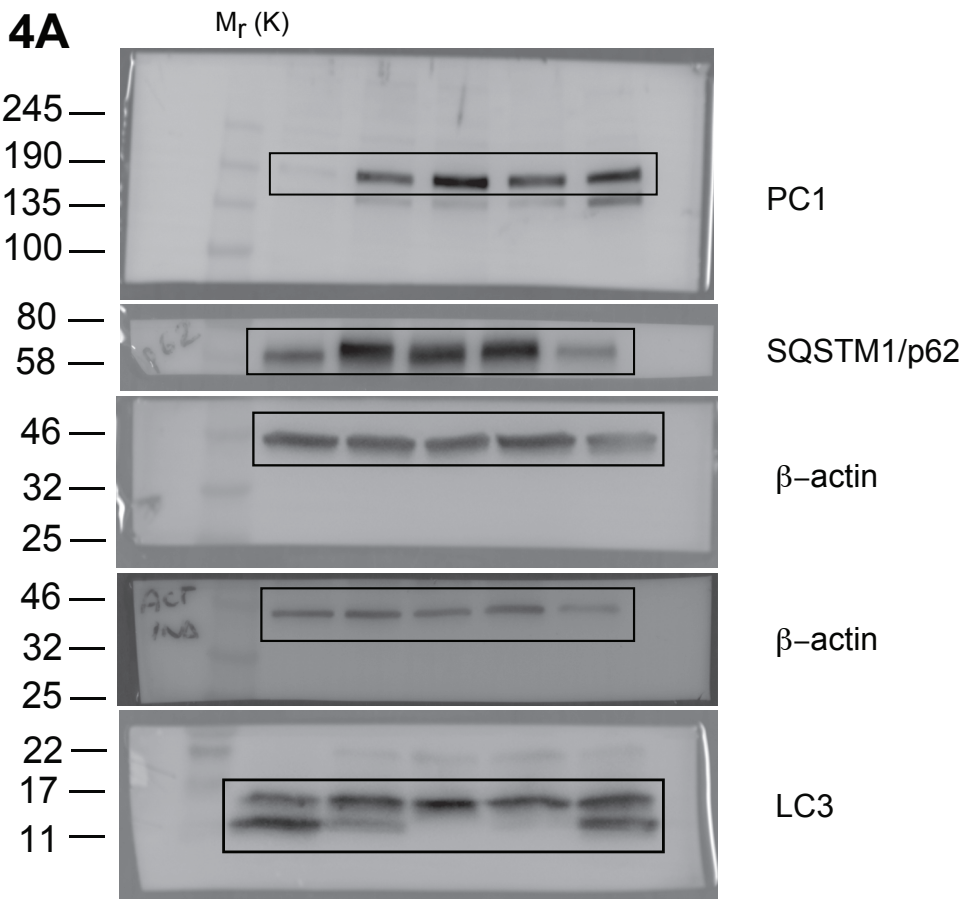

EV FIGURE 4B

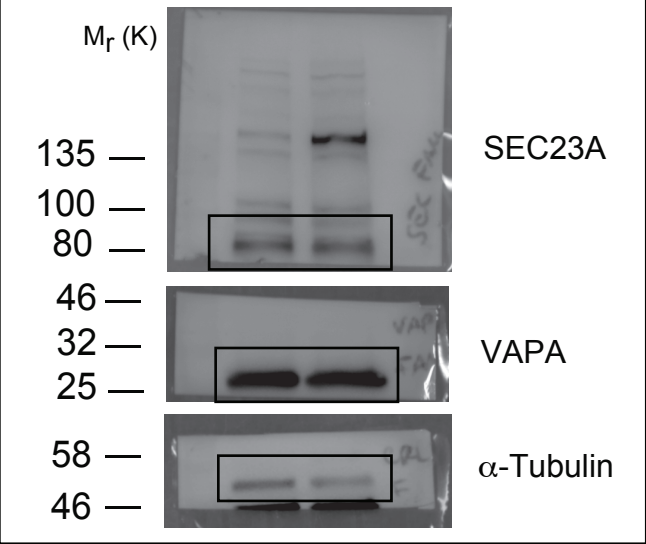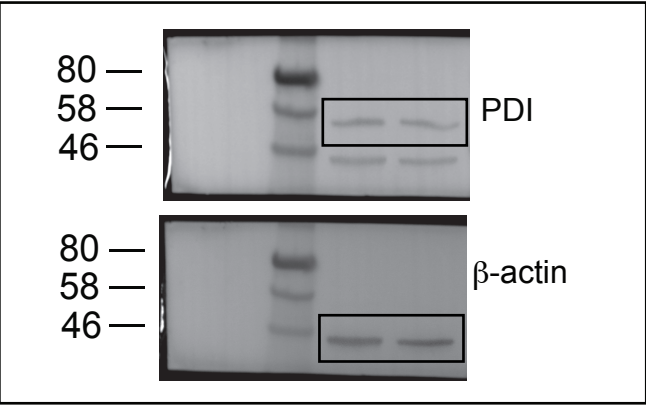

EV FIGURE 4C

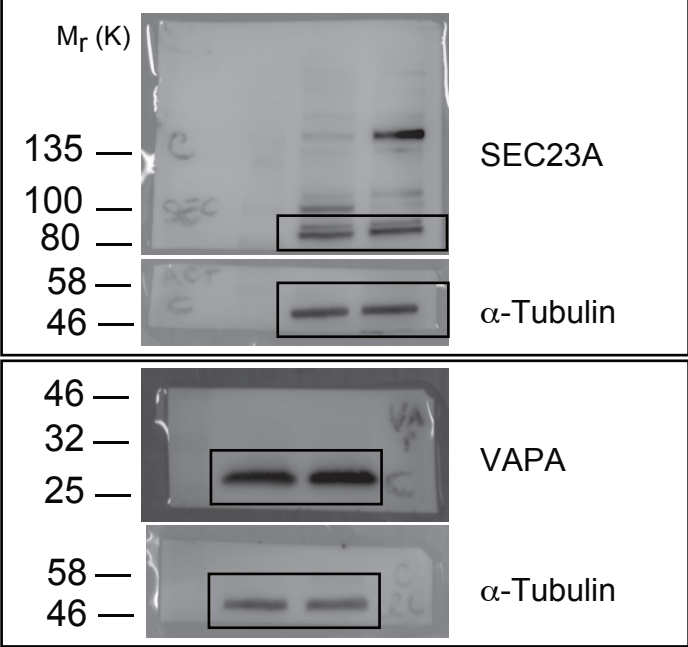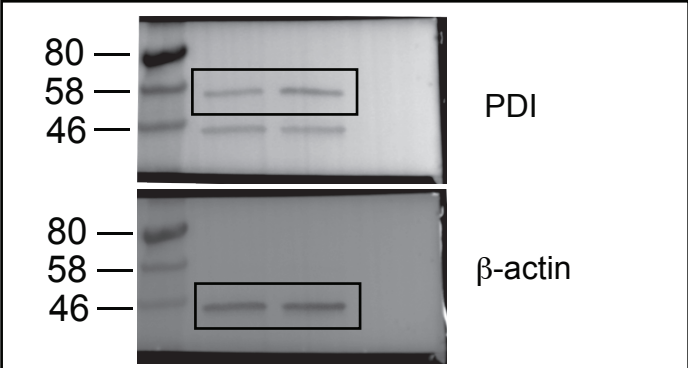

Supplement: Supplementary file 5 — Source Data for Expanded View [file EMBJ-38-e99847-s005.zip › Figure_Source_Data_Fig_EV4.pdf]

Uncropped gels with size marker indications

EV FIGURE 5A

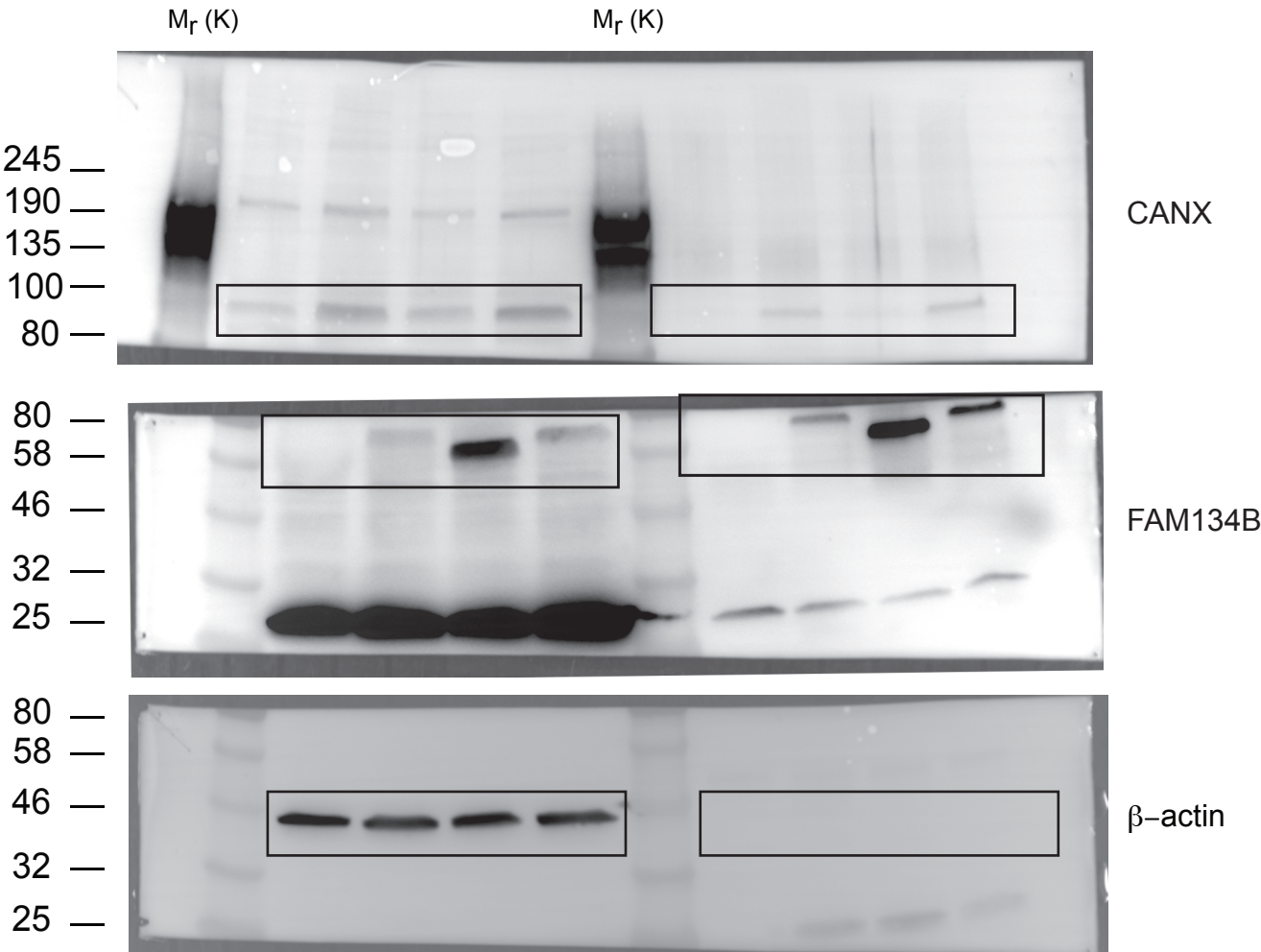

Supplement: Supplementary file 5 — Source Data for Expanded View [file EMBJ-38-e99847-s005.zip › Figure_Source_Data_Fig_EV5.pdf]

Uncropped gels with size marker indications

**FIGURE 1J**

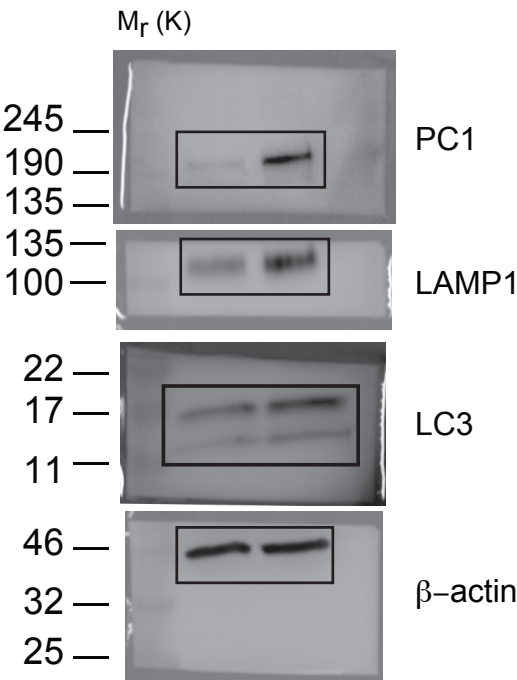

Supplement: Supplementary file 7 — Source Data for Figure 1 [file EMBJ-38-e99847-s006.pdf]

Uncropped gels with size marker indications

**FIGURE 4A**

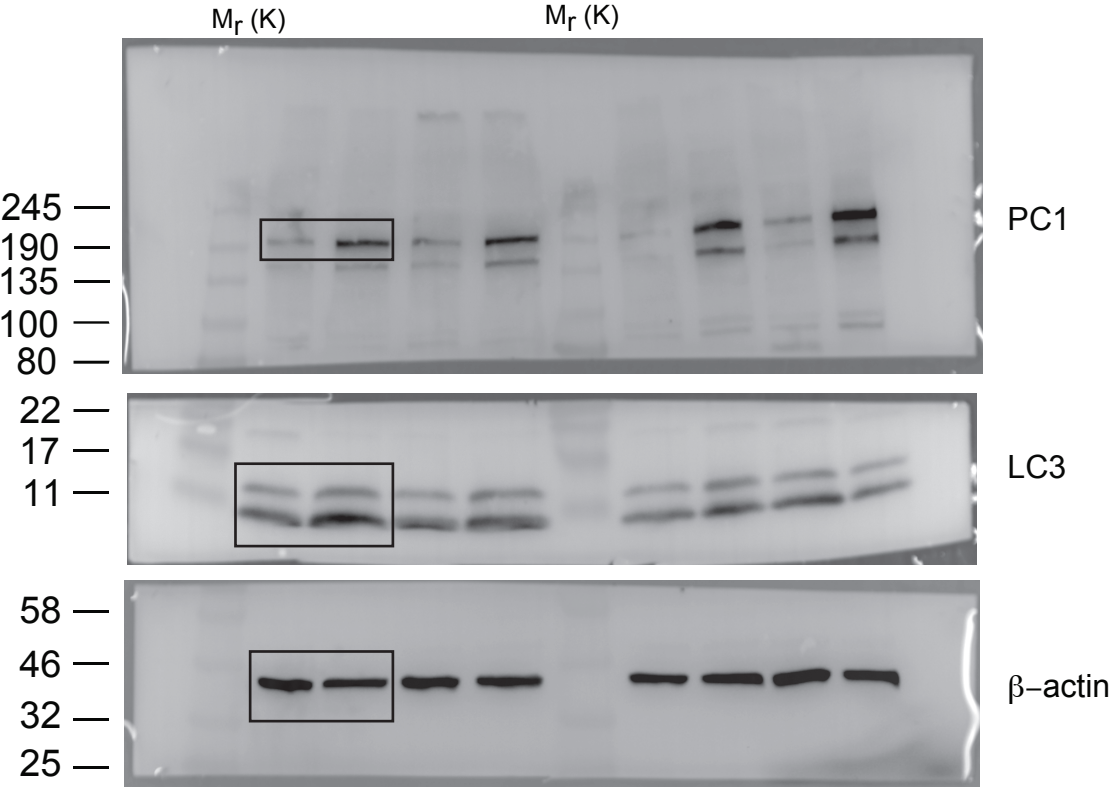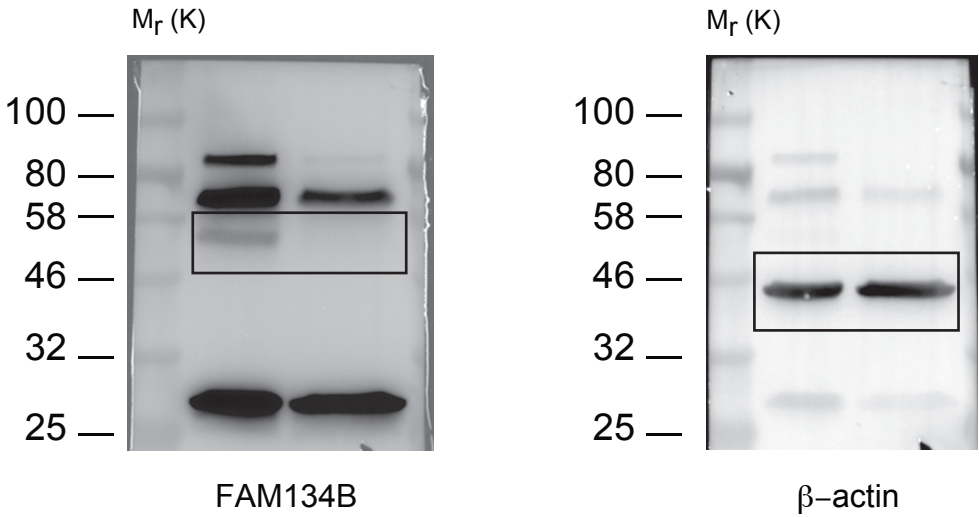

Supplement: Supplementary file 8 — Source Data for Figure 4 [file EMBJ-38-e99847-s007.pdf]

Uncropped gels with size marker indications

**FIGURE 5B**

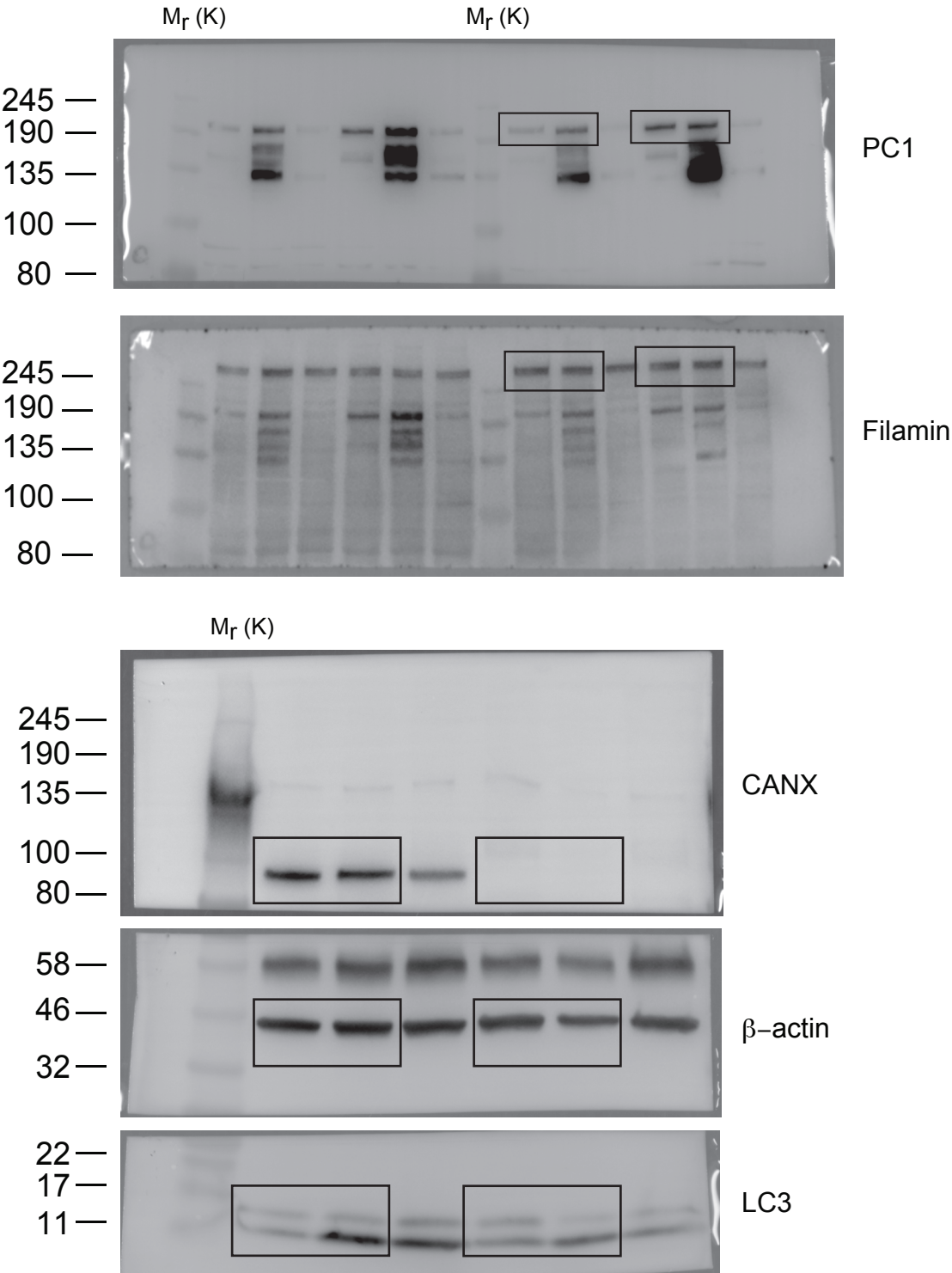

Supplement: Supplementary file 9 — Source Data for Figure 5 [file EMBJ-38-e99847-s008.pdf]

Uncropped gels with size marker indications

FIGURE 7B

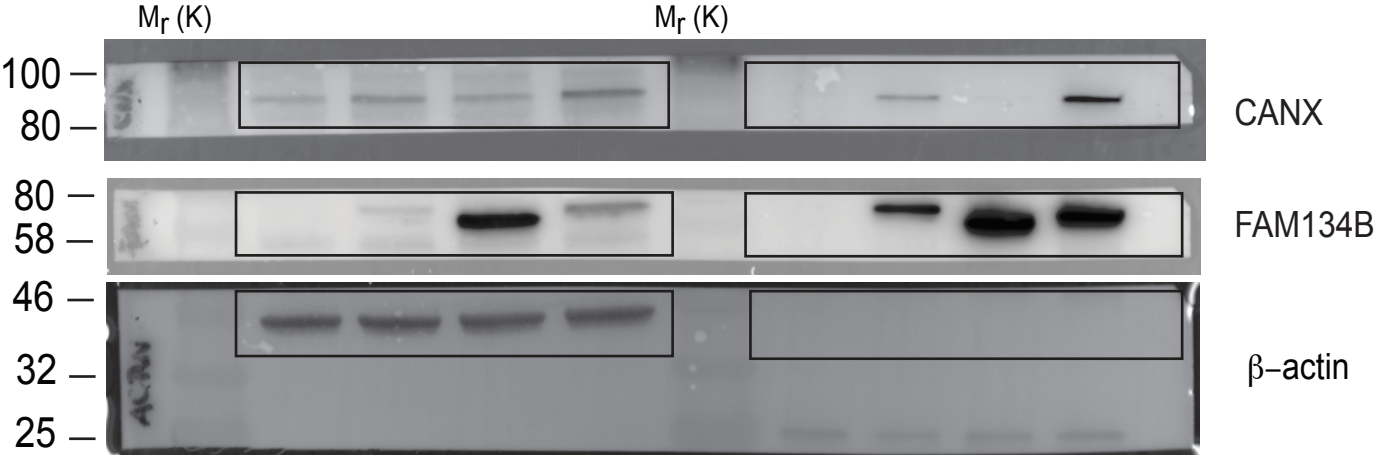

FIGURE 7C

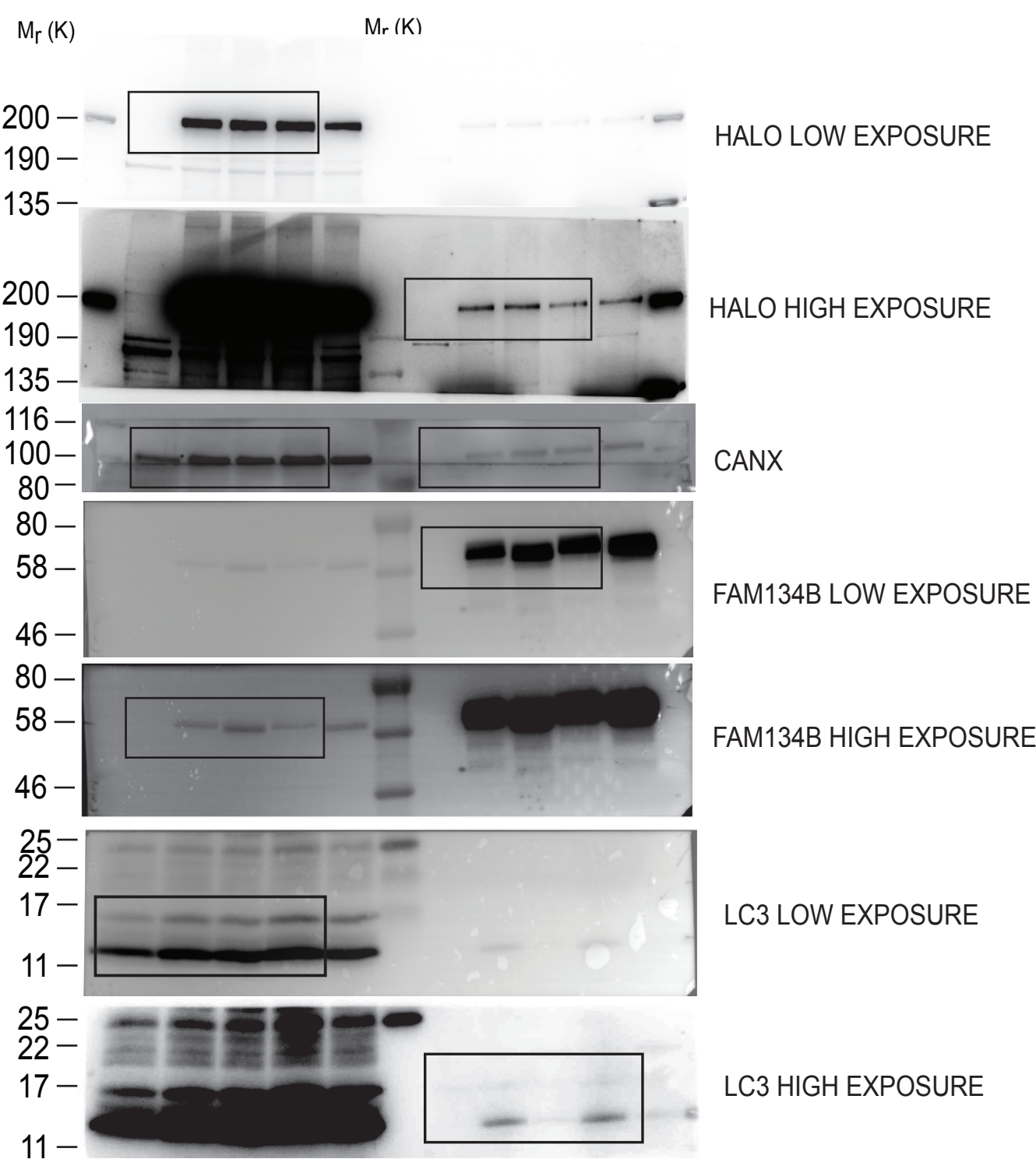

Supplement: Supplementary file 10 — Source Data for Figure 7 [file EMBJ-38-e99847-s009.pdf]
